# Correlations between the Visual Apparatus and Dental Occlusion: A Literature Review

**DOI:** 10.1155/2018/2694517

**Published:** 2018-07-09

**Authors:** Alberto Baldini, Alessandro Nota, Silvia Caruso, Simona Tecco

**Affiliations:** ^1^University of Rome Tor Vergata, Italy; ^2^Dental School, Vita-Salute San Raffaele University, Milan, Italy; ^3^MeSVA Department, University of L'Aquila, Italy

## Abstract

**Background:**

The development of visual functions takes place in the first months of postnatal life and is completed around the one year of age. In this period, the maturation of the retina and the visual pathways occur, and binocular bonds are established at the level of the visual cortex. During this phase and then for a few years, a certain plasticity of the visual functions remains, which seem therefore susceptible to change both in a pejorative sense (by pathogens) and in an improving sense (for example, by therapeutic measures). This plasticity involves also the oculomotor system. Due to this plasticity, many researchers believe that there are some functional correlations between the visual and the stomatognathic apparatus. But the scientific evidence of this statement has not been clarified yet.

**Aim:**

The purpose of this review is therefore to analyze the clinical data in this field and finally to establish their level of evidence. Studies have been collected from the main databases, based on keywords.

**Results:**

The results showed a middle level of evidence since most of the data derive from case-control studies and cross-sectional studies.

**Conclusions:**

The level of evidence allows establishing that there is a correlation between ocular disorders (myopia, hyperopia, astigmatism, exophoria, and an unphysiological gait due to ocular convergence defects) and dental occlusion, but it is not possible to establish the cause-effect relationship. Future studies should be aimed at establishing higher levels of evidence (prospective, controlled, and randomized studies).

## 1. Introduction

The visual apparatus consists of equal and symmetrical organs located, for the great part in the anterior region of the head, below the forehead, and at the sides of the nose root [1].

Among these organs, the main one is the eye; the others, called accessory organs of the eye, can be grouped into the oculomotor system, and a protective structure.

The oculomotor system allows moving the eye towards certain sectors of the environment; its particular organization and its connections with the brain make it suitable to transmit to the analytical neurological centres a complex of signals that faithfully repeat the images of the external world [1].

The eyeball, together with the oculomotor system and the protective apparatus, is located in the orbital cavity that is a deep, bone cavity, formed by the convergence of the processes of the maxillary, the zygomatic, and the palatine bones, in the lower part; by the convergence of the frontal and the sphenoid bones superiorly; from the lacrimal ethmoid and, minimally, maxillary, and sphenoid bones, medially; and from the zygomatic and the sphenoid bones, laterally [1].

The eyeball is contained in a connective structure called Tenon's capsule, which separates it from the orbital fat. It forms a sort of bulb support, for which it has the function of an acetabulum.

The development of visual functions begins in the first months of life and is completed around the one year of age [2].

In this period, the maturation of the retina and the visual pathways occur, and binocular bonds are established at the level of the visual cortex. During this phase of maturation and then—for a few years—a certain plasticity of visual functions remains; therefore, during this “plastic” period, it seems susceptible to change both in a pejorative sense (by pathogens) or in an improving sense (for example, by therapeutic measures). This plasticity appears maximum during the first months of life and then gradually decreases until it disappears around the 7th-8th year of life [2].

Moreover, during the “plastic” period of the visual system, the development of its various functions does not occur independently; as a consequence, if a pathological cause alters the development of one of the functions, the other functions may also be affected. In particular, the mechanisms of the motor and the sensory fusions, which occur in the oculomotor system during the first years of life, play an important role.

The term “sensory fusion” indicates the unification of visual excitations from the corresponding retinal images, into a* single* visual perception (a single image). A subject is unable to see double, if the external object stimulates the retinal points that have the same spatial location, defined as “correspondent” [2, 22].

A “fusionable” stimulus—different from a stimulus that could elicit a retinal rivalry—can appear from the age of 4-5 months of a child [2, 22].

The critical period in which the sensory fusion could be compromised lasts until the 6th-7th year of life. During the first two or three years of life, a few weeks of interrupted binocularity is a period able enough to definitively abolish the sensory fusion. After this period, the ocular function gradually tends to become stronger, so that at 5-6 years it could be recovered even after a long period of interruption [2, 22].

The term “motor fusion” indicates the ability to align the eyes in order to maintain the “sensory fusion”. The stimulus for these fusing eye movements is the retinal disparity. The retinal disparity means the simultaneous stimulation of mismatched retinal elements [2].

In normal conditions, the retinal image disparity produces diplopia. But the fusional movements can trigger a “vergency response” to align the images of the object in regard to the foveas. A positive fusional vergence measures the extent to which a person can maintain the “fusion” and align the eyes, with gradually increasing vergence demands.

In a normal person, the ability to recognize incorrect binocular alignments is limited by the quality of sensory information. In the first 3-4 months of life, the reduced visual acuity, the absence of stereopsis, and the immaturity of cortical neurons greatly limit the movements of fusional convergence [22]. Binocular alignment is therefore very unstable in the first months of life and becomes more accurate around the 5th or 6th month. The mechanism of fusional convergences is variously susceptible to any pathogenic factor up to about 9 years of age of the child [22].

The visual function is strongly interconnected with other functions aimed at the orientation of the individual in the surrounding space. Among these, there are, for example, the positioning of the head with respect to the cervical spine, for the maintenance of an upright posture, and the coordinated movements of the head and eyes for the exploration of the surrounding space. These interconnections are achieved through neurological links, which also involve the stomatognathic apparatus.

Several studies have shown that the stimulation of extraocular muscles induces effects on oculomotor function, and vice versa. For example, the oculomotor function is closely related to information coming from the cervical tract, through the* oculocephalic reflex*, mediated by afferents from C2 and C3 nerves. In the* oculocephalic reflex*, there is the presence of a triggering* ascending* stimulus, from the trigeminal nucleus, that carries information to the ocular nuclei and a* descending* stimulus that carries information to the spinal cord through the medial longitudinal fascicle [23]. Consequently, the simultaneous management of visual and vestibular afferent stimuli in the central nervous system influences the proper position of the head in space, according to the position of the eyes, and to the cervical posture.

Buisseret-Delmas et al. in an experiment, by injecting peroxide into the ocular muscles, observed its diffusion in many areas of the nervous system: the Gasser ganglion, the trigeminal spinal cord, and the cervical spine dorsal horn (C1-C2) [24].

A major station for these interconnections is represented by the hypoglossal nerve (XII cephalic nerve) that also gives the motor impulses to the tongue. The hypoglossal nerve is classically considered a pure motor nerve, but it also contains a proprioceptive component. During its extracranial decorsus, it receives the proprioceptive fibres from the cervical plexus (the nerves of the first three cervical vertebrae, C1, C2, and C3 nerves), thus collecting the proprioception information from the suboccipitals, trapezius, and sternocleidomastoid muscles and from the oculomotor apparatus, besides that of the tongue: consequently, this neurological connection represents a conjunction among the stomatognathic apparatus, the oculomotor system, and the cervical area.

Another major station is represented by the superior colliculus (SC), a structure which coordinates visual, somatic, and auditory information by directing the movements of the head and the eyes towards the source of a stimulus. Among the seven layers that constitute the SC, there are three sensitive maps (a visual map, a somatic map of the body surface, and a map of the spatial location of the sounds), plus a motor map. Consequently, the role of the visual function, in maintaining the balance of the head, or during walking and during all other motor coordination, is related to this structure. About this structure, it was observed in rats that neurons in the trigeminal mesencephalic nucleus (5me) can project to the SC. In addition, the existence of SC projections to neurons in 5me was reported. In particular, it seems that the SC fibres contact "en passant" small as well as large cell bodies of 5me neurons. These pathways suggest a role of the 5me neurons in oculomotor control and associated orofacial functions [25].

The cells of the 5me are called protoneurons (i.e., neurons of a neuronal chain) constituting the equivalent of a peripheral sensitive ganglion [8] that would explain the “sensitivity” of the stomatognathic apparatus to descending (stress, anxiety, etc.) and ascending stimuli (proprioception of the column, legs, and feet) [26–29].

In addition, the oculi extrinsic muscles, masticatory muscles, dental pulp, and periodontal ligaments seem linked by the 5me to the cerebellum, the reticular formation, the vestibular nuclei, the predominant nucleus, and the spinal motoneurons. The core of these connections constitutes an important centre for calculating the position and the eye movements due to connections with vestibular nuclei, the oculomotor nuclei, and the cerebellum.

On the base of all these concepts, the relationship between the oculomotor and the stomatognathic apparatus is an increasingly interesting subject for researchers in the field of dentistry.

On the base of these observations, a role for trigeminal afferents on body posture was hypothesized, but this has not yet been demonstrated conclusively [3, 4, 8].

Thus, the aim of this review was to clarify the level of evidence in the scientific literature about the correlations between the visual and the stomatognathic apparatus in human subjects.

## 2. Materials and Methods

In this systematic review, the results of relevant studies are summarized. Data were searched in the PubMed database, Scopus database, and the Cochrane Library using the following keywords: oculomotor, stomatognathic, dental occlusion, correlations, visual defects, vision problems for published or in press studies dated through June 2018. The search strategy is summarized in Table 1.

A hand research was conducted in the reference list of the resulting studies. Based on the titles, abstracts, and full texts of the studies when needed, two researchers dealt with the selection of the studies according to the following criteria: studies on human subjects, no case reports, and studies in English, Italian, Spanish, French, or German. From each of the selected studies, only the results concerning the correlations between the visual function and stomatognathic apparatus are reported. Data on the type and the construction of the study (the design, the presence of a control group, the mechanism of randomization, the method error study, and the technical description of the methods) were also recorded in order to assess their level of evidence. The quality of the studies was established by the assignment of scores to each full-text article included in the qualitative analysis. The quality of each study, with a maximum possible score of 11, was considered as follows: low: total score ≤ 4; medium: 5 ≤ score ≤ 8; high: score ≥ 9.

## 3. Results

Out of the total number of 64 articles, 19 were qualified for the final analysis.

The quality level of the studies was judged to be low for 3 studies, medium for 13 studies, not classified for 2 studies, and high for 1 study (Table 2).

Thus, the overall level of the studies on this topic is judged to be merely adequate. The level of quality is mainly affected by the lack, actually in literature, of an adequate number of prospective longitudinal reports that could really clarify the relationship between the visual system and the stomatognathic apparatus.

The main results and methods are summarized in Table 3.

A part of the studies pointed on the* anatomical contiguity* between the stomatognathic and visual apparatus. Two of these studies are virtual studies on finite elements analysis [3, 4], and there is only one longitudinal prospective study [5].

In summary, these studies emphasize the role of the sphenoid bone for the contiguity between the two areas, because it forms part of the orbit and also is in relation to the maxillary bone [3, 4].

The higher scientific evidence is given by the only prospective longitudinal study by Habeeb [5] that showed a statistically significant increase in the ocular interaxial distance between the two eyes of about 0.25 mm after rapid palatal expansion.

Another part of the studies focuses on the* neurological* connections between the stomatognathic and the visual apparatus.

These studies are mainly organized as case-control or cross-sectional observational studies [6, 8–21]. Most of the studies are on children [6, 10–21]. Some studies are on adults, mainly focusing on subjects with temporomandibular disorders [8, 9].

## 4. Discussion

The purpose of this study was to review the existing literature data on the correlations between the stomatognathic and the visual apparatus and finally to establish the level of evidence. Studies have been collected from the main databases, based on keywords.

A part of the studies pointed on the* anatomical contiguity* between the stomatognathic and visual apparatus, and other studies focus on* neurological connections*.

It is known that the eyeball, together with the oculomotor system and the protective apparatus, is located in the orbital cavity that is a deep, bone cavity, formed by the convergence of the processes of the maxillary, the zygomatic, and the palatine bones, in the lower part; by the convergence of the frontal and the sphenoid bones superiorly; from the lacrimal ethmoid and, minimally, maxillary, and sphenoid bones, medially; and from the zygomatic and the sphenoid bones, laterally [1].

The eyeball is thus contained in a connective structure called Tenon's capsule, which separates it from the orbital fat. It forms a sort of bulb support, for which it has the function of an acetabulum.

About the anatomic contiguity, this review clarifies that there is an effect on the visual apparatus after a procedure of palatal expansion. It seems that the palatal expansion is accompanied by an increase of the stress at the sphenoid bone, which could determine an increase of the interorbital distance of 0.25 mm in children, but the clinical importance of this finding is yet to be clarified.

The evidence is given by three studies; two of them are virtual studies.

In one of them, Holberg and Rudzki-Janson [4] evaluate the stress distribution induced by a palatal expander on the sutures of the cranial base through a finite elements analysis, simulating data for children and adults, and report that the greatest stress is located in the sphenoid bone that, through the pterygoid processes, is in continuity with the jawbone. In particular, the higher stress due to the palatal expander was located in the pterygoid processes and the boreholes: the superior orbital fissure and the optic foramen seem particularly affected. In addition, it was proved by Holberg [3] that this stress could lead to alterations of the oculomotor function, through a direct mechanism—changing the position of the optical hole that attaches to the Zinn tendon ring that is the common origin of 5 oculomotor muscles—and/or through an indirect mechanism, compressing one or more orbital nerves.

Although a virtual analysis can provide only a theoretical scientific evidence of relationship, the conclusions of these studies are also supported in literature by a case-report of an adult by the experience of Lanigan and Mintz [30] that describe the paresis of the ocular nerve after a surgically assisted palatal expansion, observing by tomography that the cause of this complication was a small fracture of the sphenoid.

About the anatomical contiguity, the higher scientific evidence is given by the only prospective longitudinal study by Habeeb et al. [5] that showed a statistically significant increase in the ocular interaxial distance between the two eyes of about 0.25 mm after rapid palatal expansion (in a sample of 28 children, 17 boys and 11 girls, mean age, 9.9 years), but the clinical significance of this observation remains indefinite. A clinical consequence is that the clinician can realize that with activation of the rapid maxillary expansion appliance he/she is producing not only an expansion force at the intermaxillary suture but also forces on other structures within the craniofacial complex that may or may not be beneficial for the patient [31].

About the neurological connections, most of the studies correlate the different visual defects to specific malocclusions.

The ocular convergence defects were associated with functional mandibular lateral deviation in children [10].

The ocular motility disorders were associated with unilateral crossbite with midline deviation in children in a sample of children [14] while fusional vergence defects (exophoria) were associated with molar class II malocclusions [21].

The myopia in children was associated with the presence of crossbite [18] and with the presence of class II, division I malocclusions [16, 17]. In addition, about the myopia, a pair of case-control studies report an evident association between myopia and the tonic activity of the anterior temporalis muscle. In a case-control comparison, myopic children (a sample of 10 subjects, 7-13 years old) showed a marked difference in the tonic activity of anterior temporalis muscle at open eyes, with respect to children with normal vision (10 matched subjects) [6].

In addition, a correction of the oculomotor defect programmed upon an electromyographic control of the anterior temporalis muscle resulted accompanied by a statistically significant decrease of the tonic activity of this muscle at open eyes—almost in children with oculomotor defects and mandibular functional lateral deviation—with respect to children that received a* conventional* visual correction (case-control study) [15].

The hyperopia in children was associated with the presence of a crossbite [19] and with the presence of class I malocclusions [16, 17].

The astigmatism was associated with the presence of class I malocclusions [16, 17].

The ocular right-sided condition in children was associated with a more symmetric dental occlusion (bilateral class I or II condition) [12], with respect to the not right-sidedness condition, in a cross-sectional evaluation. The ocular right-sided condition in children was also related to a lower prevalence of crossbites on the right side, with respect to the condition with non-right-sidedness in their function [13].

The presence of an unphysiological gait due to ocular convergence defects was associated with increased overjet and overbite [20].

A pair of studies focused on subjects with temporomandibular joint disorders (TMD) in the adult population, and some associations were also observed between TMD and oculomotor function in adults. Furthermore, a considerably higher prevalence of ocular convergence defects was assessed in adults affected by TMD presenting limited maximal opening, myofascial pain, and pain in the neck shoulder area, with respect to healthy individuals [9].

In addition, in a case-control study on 50 patients affected by TMD, compared with an equal number of control subjects, it was observed a significantly lower binocular function, measured as convergence and positive fusional vergence, with respect to control healthy subjects with normal disc position [8].

But the higher scientific evidence is related to a prospective controlled clinical study conducted by Sharifi Milani and Coll. [7], which reports a correlation between dental occlusion and visual focus in children treated with a jaw orthopaedic repositioning device. This device, especially in children with occlusal disarming, caused changes in the focus ability that disappeared by removing the device itself. In this study thirty subjects were divided into two groups: an experimental group treated with mandibular orthopaedic repositioning appliances and an untreated control group. All of the subjects underwent the same visual focusing tests with a Maddox rod and the Berens prismatic bars, from over five meters to 30 centimetres. The results showed that the alteration of dental occlusion can induce some fluctuations in visual focusing. The phenomenon occurred after wearing a mandibular orthopaedic repositioning appliance for a while. Feedback effects were gradual after removing the mandibular splint.

As the great part of the studies are based on a case-control or a cross-sectional construction, only some hypotheses were possible to explain the reasons for these types of correlations.

The great part of the researchers seem in agreement to assume that the nociceptive stimuli due to the inflammation of masticatory muscles or temporomandibular joint following malocclusion in children may cause some alterations in nerve conduction (such as hyperexcitability, temporal aggregation, and glial cell activation) so as to determine a “central fatigue” and therefore induce recruitment of fewer muscle fibres for eye movements: this could explain the alteration of binocular mobility [32].

These assumptions seem to be supported by a case-control study published by Erturk and Dogan [11] that compared craniofacial and dentoalveolar morphology in blind children and control healthy children. Their results revealed a change in the inclination of the head and a reduction in the size of the upper jaw in blind children, the latter probably related to the insufficient development of the orbital region that was detected. The same authors also hypothesized a correlation between the crown dimension of deciduous teeth and strabismus and suggested that strabismus can result from child developmental changes and that the resulting asymmetries may involve the whole head, including the teeth.

## 5. Conclusions

The purpose of this study was to review the existing literature data on the correlations between the stomatognathic and the visual apparatus and finally to establish the level of evidence.

As the great part of the studies in this field are observational case-control or cross-sectional analyses, their level of evidence allows establishing that there is a correlation between ocular disorders (myopia, hyperopia, astigmatism, exophoria, and an unphysiological gait due to ocular convergence defects) and dental occlusion, but it is not possible to establish the cause-effect relationship.

Furthermore, in adult samples, a considerably higher prevalence of ocular convergence defects was assessed in adults affected by TMD.

A slight evidence, based on very few data, allows us to establish that intraoral appliances could change ocular ability: it seems that there is an increase of ocular interaxial distance between the two eyes of about 0.25 mm after a rapid palatal expansion in children; and in addition a mandibular anterior repositioning appliance, especially in children with occlusal disarming, seems to cause changes in the focus ability that disappear by removing the device itself.

But these evidences are based only on small samples.

Consequently, future studies should be aimed at establishing higher levels of evidence (prospective, controlled, and randomized studies).

## Figures and Tables

**Table 1:** Search strategy.

1	Oculomotor

2	Stomatognathic

3	Dental occlusion

4	Visual defects

5	Vision problems

	1 or 4 or 5 and 2 or 3

**Table 2:** Qualitative analysis of the studies.

	Authors	Type of study	Sample selection adequacy based on age range across the group/s	Sample selection adequacy based on gender across the group/s	Description of at least an error analysis method	Complete description of technical data	Description of blinding procedure	Prior estimation of sample size or a posteriori power analysis	Points
		Longitudinal Construction: 2 Other methods: 1 Not defined: 0	Full: 2 points: properly and clearly detectable data according to age range (i.e., data derived from groups of children or adolescents, or young adults, or adults) Partial: 1 point Not: 0 points	Full: 2 points Balanced distribution of males and females and separate reports for males and female Partial: 1 point No: 0 points	Yes: 1 point No: 0 points	Complete: 2 Partial: 1 Insufficient: 0	Yes: 1 point No: 0 points	Yes: 1 point No: 0 points	

1	Holdberg [3]	1	0	0	0	0	0	0	n.c.

2	Holdberg and Rudzki-Janson [4]	1	0	0	0	0	0	0	n.c.

3	Habeeb et al. [5]	2	2	1	0	2	0	0	7

4	Monaco et al. [6]	1	2	1	1	2	0	0	7

5	Sharifi Milani et al. [7]	2	2	1	1	2	1	0	9

6	Cuccia and Caradonna [8]	1	2	1	0	2	0	0	6

7	Monaco e al. [9]	1	2	1	1	2	0	0	7

8	Monaco et al. [10]	1	2	1	1	2	0	0	7

9	Erturk et al. [11]	1	1	1	0	1	0	0	4

10	Heikkinen et al. [12]	1	2	0	0	1	0	0	4

11	Heikkinen et al. [13]	1	2	0	0	1	0	0	4

12	Bollero et al. [14]	1	2	1	0	2	1	0	7

13	Monaco et al. [15]	2	2	1	1	2	1	0	9

14	Monaco et al. [16]	1	2	1	0	2	0	0	6

15	Monaco et al. [17]	1	2	1	0	2	0	0	6

16	Monaco et al. [18]	1	2	1	0	2	0	0	6

17	Monaco et al. [19]	1	2	1	0	2	0	0	6

18	Silvestrini-Biavati et al. [20]	1	2	1	0	2	0	0	6

19	Caruso et al. [21]	1	2	1	0	2	0	1	7

**Table 3:** Main results of the studies.

	**Article**	**Year of publication**	**Type of article**	**Sample (number of subjects)**	**Range of age **	**Control group** **(yes/not)**	**Randomization** **(yes/not)**	**Mean results**
1	C. Holberg [3]	2005	Virtual experiment: A finite element model of the sphenoid	/	/	/	/	During palatal expansion, due to lateral bending of the pterygoid processes, marked stress develops in the round and oval foramen regions and those of the superior orbital fissure, where fractures causing neural and vascular injury can occur

2	Holberg and Rudzki-Janson [4]	2006	Virtual experiment: Cranial constructions from the CT data (computerized simulations)	/	/	/	/	The superior orbital fissure and the optic foramen seem particularly affected by stress during the rapid palatal expansion procedure

3	Habeeb et al. [5]	2013	Prospective clinical study	28 children (17 M; 11 F) (mean 9.9 years)	7.8-12.8 years	/	/	An increase in the ocular interaxial distance between the two eyes of about 0.25 mm after treatment was reported in children who required rapid palatal expansion

4	Monaco et al. [6]	2006	Case-control study	10 myopic children + 10 subjects, matched by gender and age	7-13 years	Yes	/	There is a marked difference in tonic activity of temporal anterior muscles at open eyes between the myopic and the normal children

5	Sharifi Milani et al. [7]	1998	Prospective controlled clinical study	15 subjects, who had worn mandibular orthopaedic repositioning appliances + 15 subjects, matched by gender and age		Yes	No	After wearing a mandibular orthopaedic repositioning appliances for a while, there are some fluctuations in visual focusing

6	Cuccia and Caradonna [8]	2008	Case-control study	50 symptomatic subjects with bilateral TMJ disc displacement (13 M, 37 F; mean age, 28.84 +/- 8.22 years) + 50 asymptomatic volunteers with normal disc position (14 M, 36 F; mean age, 29.96 +/- 5.04 years).	18-40 years	Yes	/	Subjects with TMJ disc displacement had a statistically significant higher alteration in binocular function (reduction in convergence, and positive fusional vergence) compared with control subjects with normal disc positions

7	Monaco et al. [9]	2003	Case-control study	48 subjects (12 M and 36 F; average age 35) with Temporomandibular disorders (TMD) and muscular pain and/or dysfunction + 48 subjects, matched by gender and age	19-45 years	Yes	/	The TMD subjects presented a higher statistical percentage of ocular convergence defects, with respect to control subjects

8	Monaco et al. [10]	2004	Case-control study	60 children with functional mandibular latero-deviation + 60 healthy children matched by gender and age.		Yes	/	In children affected by mandibular laterodeviation, ocular convergence defects occurred in greater frequency with respect to control children

9	Erturk and Dogan [11]	1990	Case-control study	20 blind subjects, who had been totally blind since birth + 20 normal-sighted subjects, matched by gender and age		Yes	/	Bind children show a significant difference in head posture, and in craniofacial and dentoalveolar morphology: an increase in the mandibular angle and in vertical jaw relationships and a decrease in inclination of the mandibular incisors, with respect to normal-sighted persons

10	Heikkinen et al. [12]	2004	Cross-sectional study	1423 young American black and white children (with II class cusp sagittal relationship) (mean age: 8.5).	6-12 years	/	/	True right-sided children show a more symmetric occlusion (bilateral Angle I or II class) than non-right-sided children (unilateral Angle I or II class)

11	Heikkinen et al. [13]	2005	Cross-sectional study	1423 young American black and white children (with II class cusp sagittal relationship) (mean age: 8.5).	6-12 years	/	/	True right-sided children show less crossbite on the right side than children having non-right-sidedness in their functions, with the differences being statistically significant

12	Bollero et al. [14]	2017	Cross-sectional study	84 subjects (49 M, 35 F)	7.3±1.7 years	/	/	A statistically significant correlation is reported between ocular motility disorders and unilateral cross-bite with midline deviation.

13	Monaco et al. [15]	2006	Case control-study	32 children with functional lateral deviation of mandible and oculo-extrinsic muscular tone disorders, randomly divided into two groups: study and control. In the study group the ocular defects were corrected upon the support of an electromyographic control (in the control group in a conventional way)	8-12 years	Yes	Yes	Both groups showed a significant statistical increase of tonic activity at rest with eyes open in the anterior temporalis muscle. But a significant decrease of this value was observed only with ocular correction upon electromyographic control (in the study group).

14	Monaco et al. [16]	2013	Cross-sectional evaluation	1.326 patients classified as Class I, Class II, and Class III		no	no	The prevalence of myopia was higher in Class II malocclusions, while the prevalence of astigmatism and hyperopia was higher in Class I malocclusion. No significant difference in vision defects by sex was found.

15	Monaco et al. [17]	2012	Cross-sectional evaluation	292 selected subjects		no	no	A statistical significant higher prevalence was found for subjects showing myopia in Class II division 1 malocclusion, while no other significant differences were found for prevalence in the other classes of malocclusions.

16	Monaco et al. [18]	2011	Cross-sectional evaluation	176 selected subjects	39 study subjects with cross-bite + 137 control subjects without crossbite	no	no	Statistically significant correlations were found between astigmatism and cross-bite, while no associations were found with other malocclusions. No gender influence was found for astigmatism or malocclusion.

17	Monaco et al. [19]	2011	Cross-sectional evaluation	176 selected subjects	32 study subjects with hyperopia + 138 control subjects	no	no	Statistically significant correlations were found between hyperopia and cross-bite while no associations were found with other malocclusions.

18	Silvestrini-Biavati et al. [20]	2013	Cross-sectional evaluation	605 selected subjects	8-10 years	no	no	A prevalence of cases with an unphysiological gait was found in patients with overjet (14.70%) or overbite (14.87%), while the percentage of patients with normal occlusion that showed an unphysiological gait was 13.08%. Subjects with an open bite or deep bite showed a slightly different distribution of right or left dominant eyes.

19	Caruso et al. [21]	2018	Cross-sectional evaluation	36 (21M, 13F;)	12±2 years	no	no	A statistically significant association between occlusal molar class II and exophoria (78.3% of subjects with molar class II have exophoria, p<0.05) and fusional amplitudes below the cut-off value (83.3%, p<0.05).
